# Osteomyelitis caused by *Aspergillus terreus* complex in a dog: a case report

**DOI:** 10.1186/s12917-023-03628-x

**Published:** 2023-06-08

**Authors:** Raquel Abreu, António Martinho, Rute Noiva, Hugo Pissarra, João Cota, Eva Cunha, Luís Tavares, Manuela Oliveira

**Affiliations:** 1 CIISA - Centro de Investigação Interdisciplinar Em Sanidade Animal, Faculdade de Medicina Veterinária, Universidade de Lisboa, Lisboa, Portugal; 2 Laboratório Associado Para Ciência Animal E Veterinária (AL4AnimalS), Lisboa, Portugal

**Keywords:** Dog, *Aspergillus terreus* complex, Fungal osteomyelitis, Itraconazole

## Abstract

**Background:**

In dogs, the most frequently reported mycosis associated with *Aspergillus* spp. are respiratory infections*.* Systemic aspergillosis is uncommon, with reported cases been associated with several *Aspergillus* species. *Aspergillus terreus* species complex are ubiquitous organisms, unfrequently associated with local or systemic disease in animals and humans, and treatment of osteomyelitis caused by this species is usually unfavorable.

**Case presentation:**

This case report describes the case of a 5-year-old dog, referred to the Veterinary Hospital of the Faculty of Veterinary Medicine of the University of Lisbon, Portugal, with a history of lameness of the right thoracic limb. Radiographs and CT scan revealed two different lesions on right humerus and radio, which were biopsied. The samples collected were submitted to cytological and histopathological evaluation and bacterial and mycological culture. Environmental samples, including of the surgery room and of the biopsy needle were also evaluated for the presence of fungi. Regarding biopsy samples, bacterial culture was negative, but mycological analysis originated a pure culture of a fungal species later identified as *Aspergillus terreus* by Sanger sequencing. Results were compatible with histopathologic examination, which revealed periosteal reaction and invasion of hyphae elements. Also, mycological analysis of both environmental samples evaluated were negative. The virulence profile of the fungal isolate was phenotypically characterized using specific media, allowing to reveal its ability to produce several enzymes involved in its pathogenicity, namely lipase, hemolysin and DNAse, corresponding to a Virulence Index (V. Index.) of 0.43. The patient was submitted to itraconazole therapy for 8 weeks. After 3 weeks, the patient showed significant clinical improvement, and after 6 weeks no radiographic signs were observed.

**Conclusions:**

Antifungal therapy with itraconazole can contribute to the remission of canine infections promoted by *Aspergillus terreus* complex with a relevant V. Index.

**Supplementary Information:**

The online version contains supplementary material available at 10.1186/s12917-023-03628-x.

## Background

*Aspergillus* spp*.* are ubiquitous fungi of worldwide distribution, commonly found in soil, water and organic matter. They are saprophytic, but several species also have an opportunistic character, being involved in various local and systemic infections in animals and humans, especially in immunocompromised patients [1, 2].

In dogs, the most frequently reported mycosis associated with *Aspergillus* spp. are respiratory infections, involving the nasal cavity and/or the paranasal sinus, with a higher prevalence in dolichocephalic breeds [3, 4]*.* Disseminated aspergillosis in dogs is uncommon, with reported cases been associated with several *Aspergillus* species [5–12], including *A. terreus* [8, 13–18]. This saprophytic fungus has also been described as an widespread opportunist pathogen, being able to produce several virulence factors associated with evasion of the host immune system and tissue adhesion, degradation and invasion [12, 19, 20].

In dogs, *A. terreus* complex has been described as the cause of several disseminated infections, mainly involving the cardiopulmonary and skeletal system [13, 21]. Although fungal osteomyelitis is rare and mainly caused by *Candida* species [22, 23], the association of *A. terreus* complex with dog osteomyelitis has been reported [13, 14, 16, 24]. This disease, characterized by the inflammation of bone and bone marrow, usually occurs after iatrogenic or spontaneous inoculation of fungi or bacteria into traumatic or surgical wounds. It can also be caused by hematogenous inoculation, which is more frequent in juveniles than in adults, occurring in metaphysis and epiphysis in the former, and in diaphysis of long bones in the latter [23].

Fungal osteomyelitis is usually difficult-to-treat in all animal species since bone tissues are not easily penetrated by antifungals [22, 23]. When promoted by fungi such as *Aspergillus*, successful treatment options for osteomyelitis decrease and the prognosis is usually unfavorable [22, 25], with retrospective studies indicating that the *A. terreus* complex infections are refractory to amphotericin B [8, 26]. Long-term treatment with itraconazole up to three years may clear the infection or prolong the survival time [14]. Also, treatment with posaconazole appears to be safe for the treatment of disseminated aspergillosis in dogs, with prolonged treatment being associated with long-term survival over one year. However, relapse is common [25], being of special importance to eliminate and avoid the dissemination of *Aspergillus* virulent strains.

## Case presentation

A 5-year-old dog, male, mixed breed, was referred to the Veterinary Hospital of the Faculty of Veterinary Medicine of the University of Lisbon, Portugal for Computerized Tomography (CT) evaluation of right thoracic limb, due to lameness. This dog presented clinical signs associated with pain in the right thoracic limb, and radiographs had already been performed at the first opinion consultation.

**Fig. 1:**
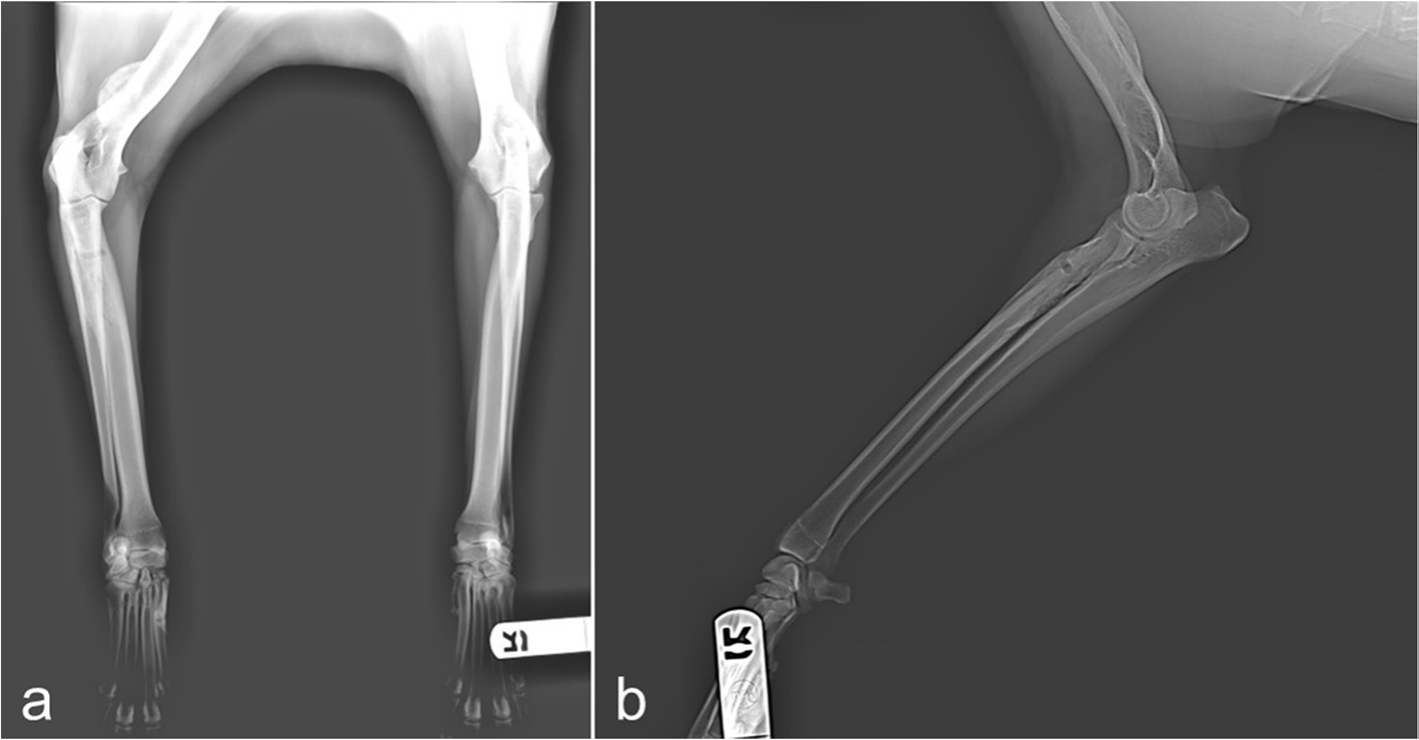
Radiographic images of the right thoracic limb. **a** Lateromedial view; **b** Anteroposterior view

A lateromedial radiograph of the right thoracic limb revealed the presence of a bone lesion at the level of the caudo-lateral diaphysis of humerus and cranial portion of the radio. The radiograph of left thoracic limb showed no alterations (Fig. 1).

**Fig. 2:**
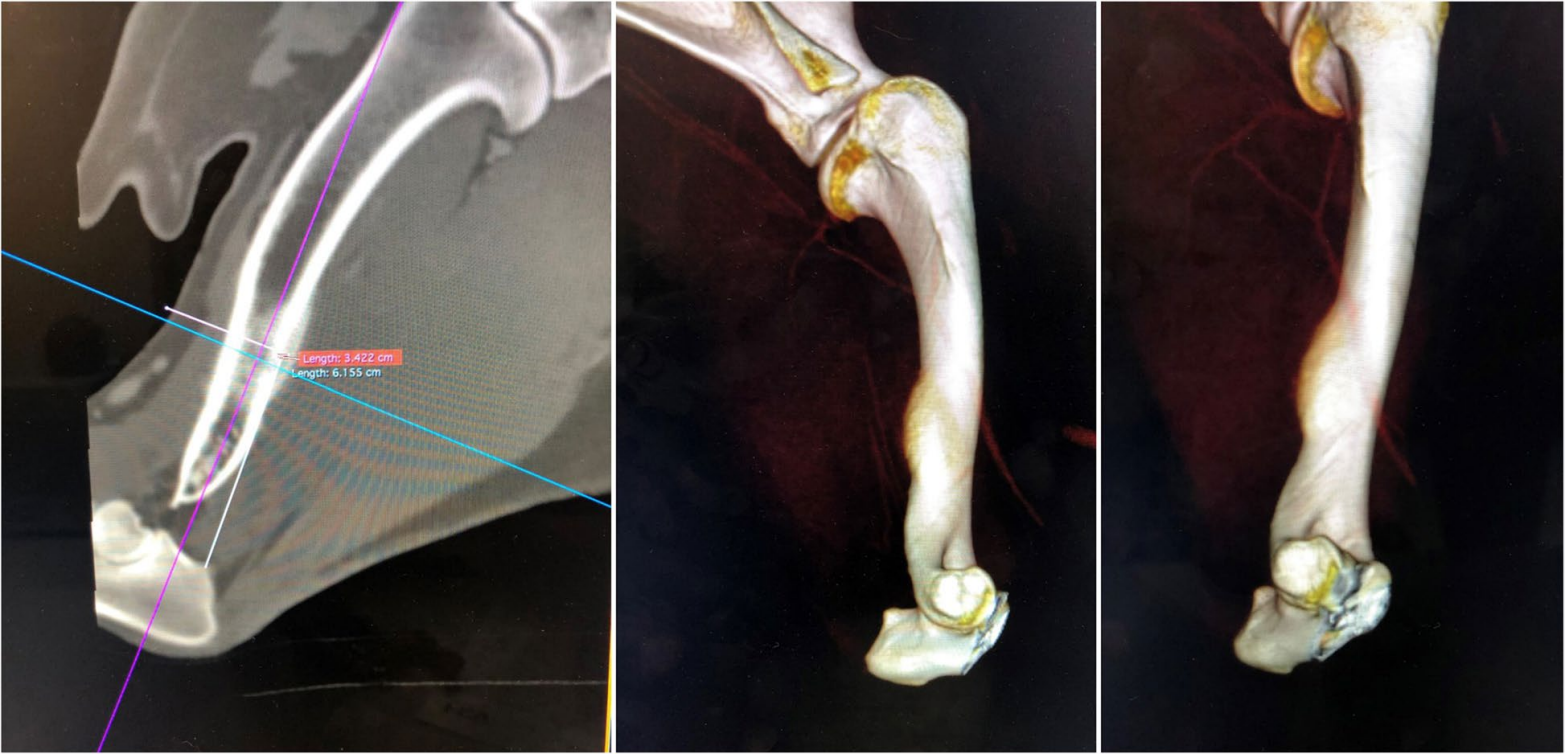
CT images of the humerus lesion

**Fig. 3:**
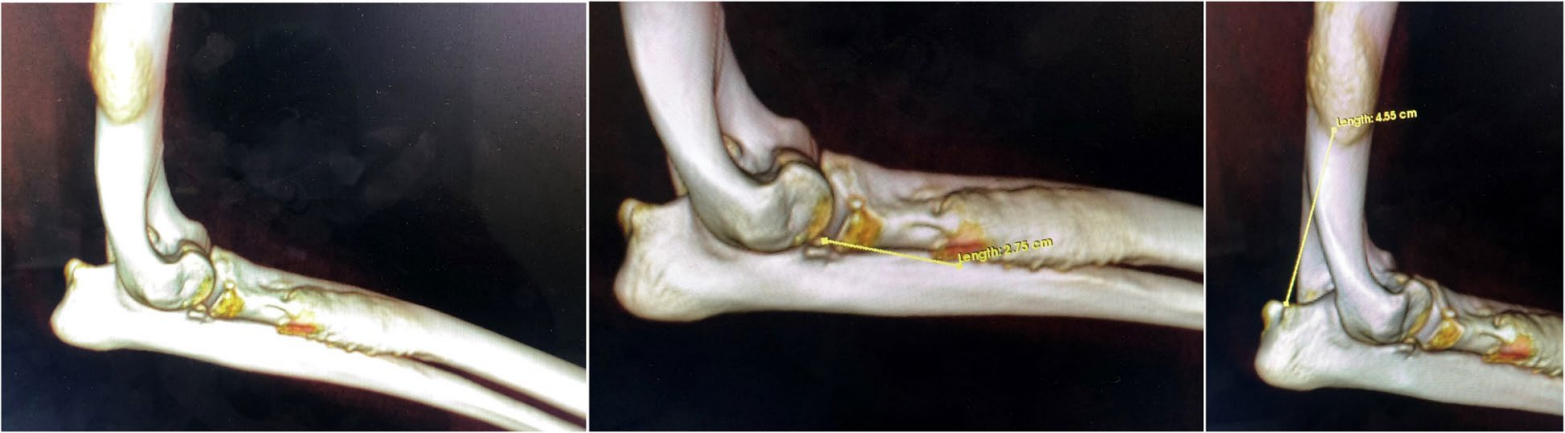
CT images of the radio lesion

Differential diagnosis, based on physical and radiographic signs, included osteolytic lesion with bacterial or fungal origin or bone neoplasia. On CT, a bone lesion was detected in the lateral and posterior side of the right humerus’ diaphysis, next to the nutrient foramen (Fig. 2). The lesion corresponded to a solid periosteal reaction, with focal enlargement of the medullary cavity, with extension of 31 mm and interruption of the bone cortical. There was a second lesion with similar traits on the proximal diaphysis of the right radio, with an extension of 45 mm (Fig. 3). There were no signs of intrathoracic, axillar or prescapular lymphadenomegaly.

Guided by CT images, fine needle aspiration and surgical biopsies were performed and material was collected for cytological and histopathological evaluation, as well as for bacterial and mycological culture. For aerobic bacterial culture, samples were inoculated in Columbia Agar + 5% Sheep Blood (bioMérieux, Marcy-l’Etoile, France), MacConkey Agar (Oxoid, Hampshire, UK) and Brain Heart Infusion Broth (BHIb) (Oxoid, Hampshire, UK), and incubated at 37 °C for 24 h. For mycological culture, samples were inoculated in Sabouraud Dextrose Agar (VWR, Leuven, Belgium), and incubated at 25 °C for 5 days.

**Fig. 4:**
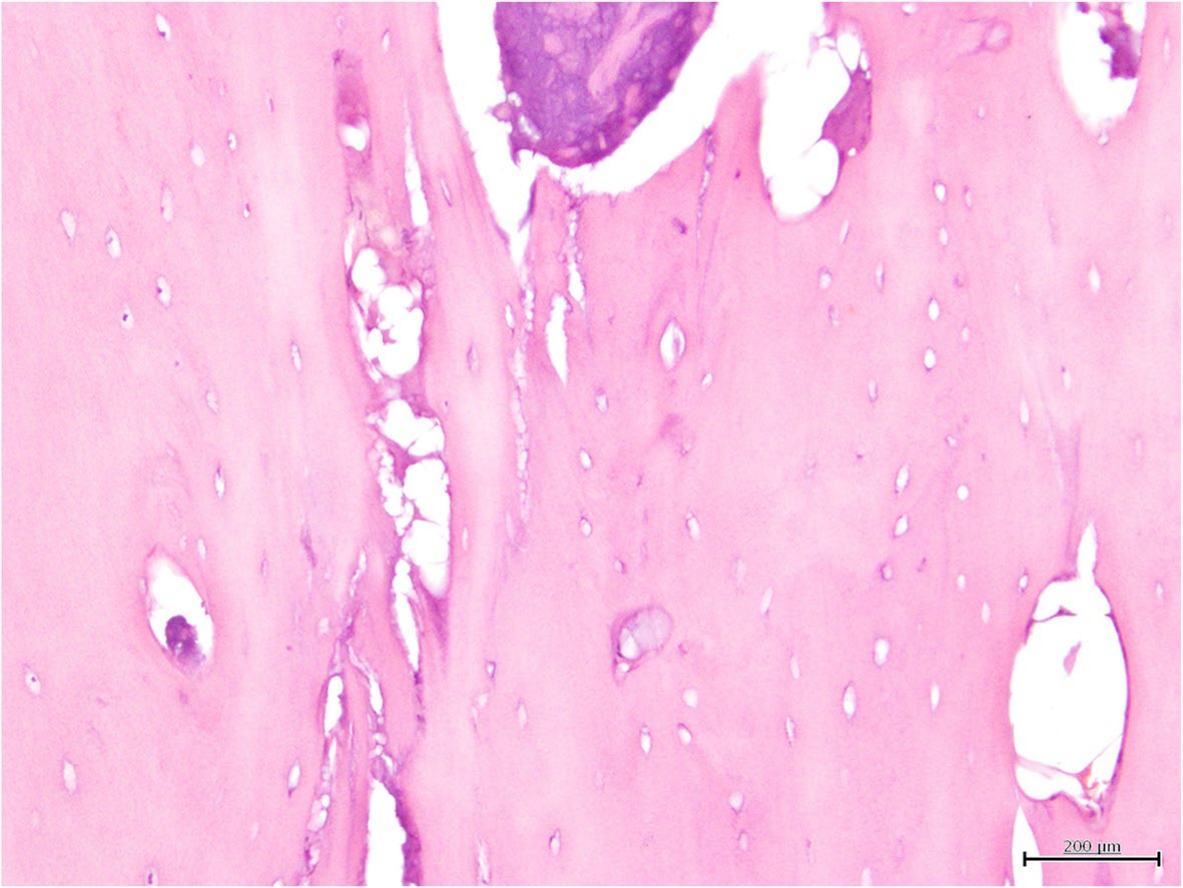
Hematoxylin and Eosin (H&E) stained histopathological preparation of a bone fragment. It is possible to observe extensive areas of fragmentation and necrosis, characterized by loss of mineralized matrix, which lost its characteristic eosinophilia and is now replaced by a basophilic to amphophilic amorphous mass (bar = 200 µm)

**Fig. 5:**
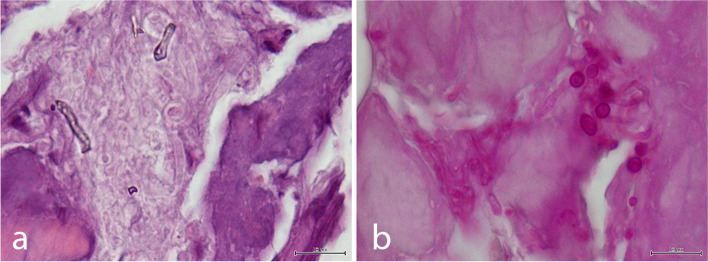
Histopathological preparations where it is possible to observe fungal invasion. **a** It is possible to observe a tangle of poorly-staining septate hyphae, with parallel, birefringent walls that branch dichotomously. Occasionally, these hyphae have bulging terminal expansions (H&E) (bar = 10 µm); **b** Special staining with Periodic Acid Schiff highlights the hyphal walls and terminal buds (bar = 10 µm)

On cytology, blood cells and scattered cells with osteoplastic and fibroblastic profile were observed, having a low grade of pleomorphism suggestive of a periosteal reaction. The histopathologic evaluation of the radio’s lesion revealed a focal chronic severe necrosis and granulomatosis osteomyelitis of mycotic origin, while the evaluation of the humerus lesion revealed periosteal neoformation (periosteal reaction) (Figs. 4 and 5).

Bacteriological analysis allowed to observe the development of cottony colonies with a brownish coloration on Columbia Agar. Microscopic observation of BHIb suspensions revealed the presence of hyphae and the absence of bacterial cells.

**Fig. 6:**
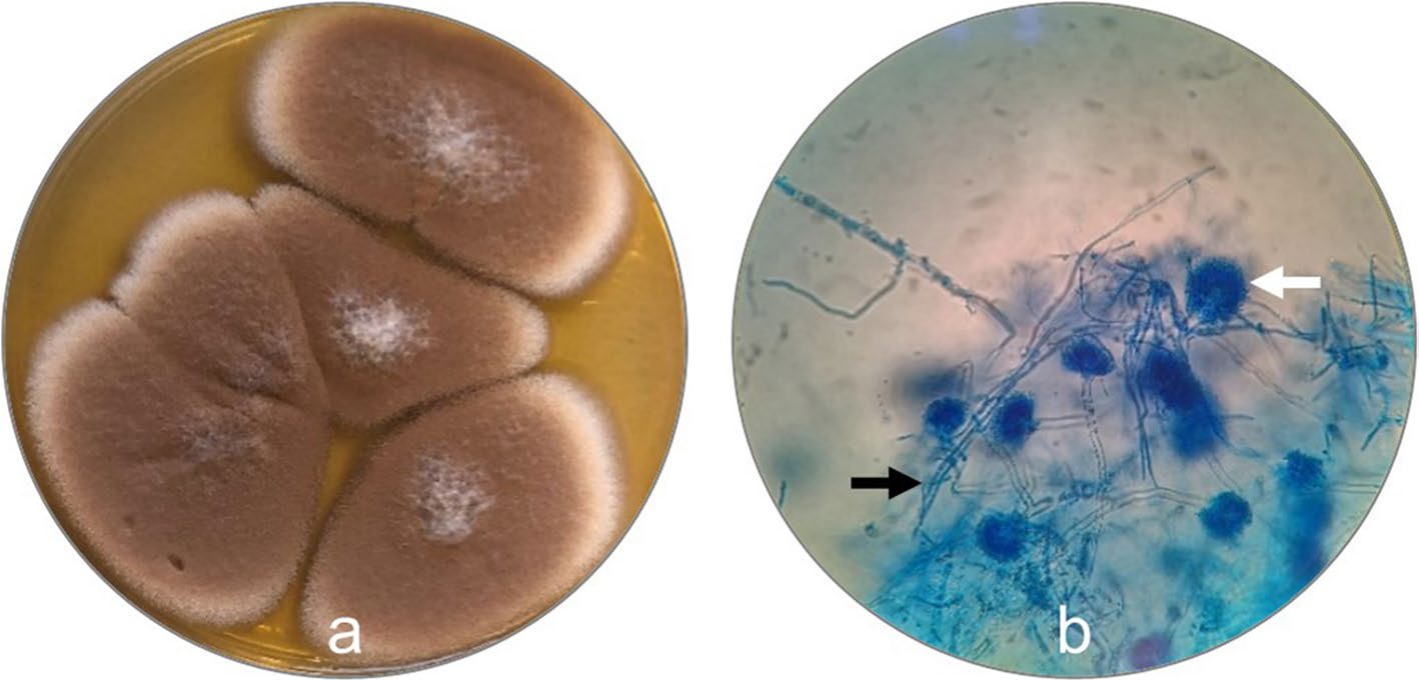
Macroscopic and microscopic morphology of *A. terreus.* **a** Pure culture of *A. terreus* in Sabouraud Dextrose Agar; **b** Microscopical presentation of *A. terreus*, showing hyaline and septate hyphae (black arrow) and hyaline conidiophores (white arrow) (LCB staining) (× 400 magnification)

In mycological cultures, an abundant growth of a pure culture presumptively identified as *A. terreus* was observed. Colonies were firstly identified through their macroscopic and microscopic traits, being characterized as brownish, white-bordered colonies, with floccose texture (Fig. 6a). The microscopic evaluation of the colonies, performed through wet mount technique using Lactophenol Cotton Blue (LCB) staining, revealed the presence of hyaline and septate hyphae, hyaline conidiophores from which biseriate phialides are formed, with round and smooth walled elliptical conidia in long chains, also compatible with *A. terreus* (Fig. 6b) [27, 28].

For confirmation of the isolate identification, fungal genomic DNA was extracted using a commercially available kit (E.Z.N.A.® Fungal DNA Mini Kit, Omega Bio-tek, Norcross), following manufacturer’s instructions. Then, DNA was amplified by conventional PCR, with a final volume mixture of 25 µL, containing 2 µL (0,8 µM) of each primer [ITS1 (5-TCCGTAGGTGAACCTGCGG) and ITS2 (5-GCTGCGTTCTTCATCGATGC)], 10 µL of DNA, 10 µL of MasterMix (NZYtaq 2 × Green, NZYtech®) and 1 µL of PCR grade water. PCR amplification was completed using the XT^96^ thermal cycler (VWR®), by applying the following conditions: 95 °C for 10 min, followed by 60 cycles of 94 °C for 15 s, 55 °C for 30 s and 72 °C for 30 s, and a final extension at 72 °C for 5 min [29]. After amplification, PCR products were separated by 1.5% agarose gel electrophoresis stained with Green Safe (NZYtech®) and visualized by transillumination (ChemiDoc XRS + , Biorad®). Afterwards, to confirm species identification, PCR products were evaluated through DNA Sanger sequencing by STABVIDA® (Lisbon, Portugal). Sequencing results confirmed the identification of the isolate as *Aspergillus terreus* (Additional files 1 and 2).

In spite of the mycological culture findings being supported by the histopathologic diagnostic, to further confirm *A. terreus* association with osteomyelitis development in this animal, two environmental samples were also evaluated, namely of the surgery room environment and of the biopsy needle, which were inoculated in Sabouraud Dextrose Agar and incubated at 25 °C for 5 days. Both samples resulted in negative fungal cultures.

**Fig. 7:**
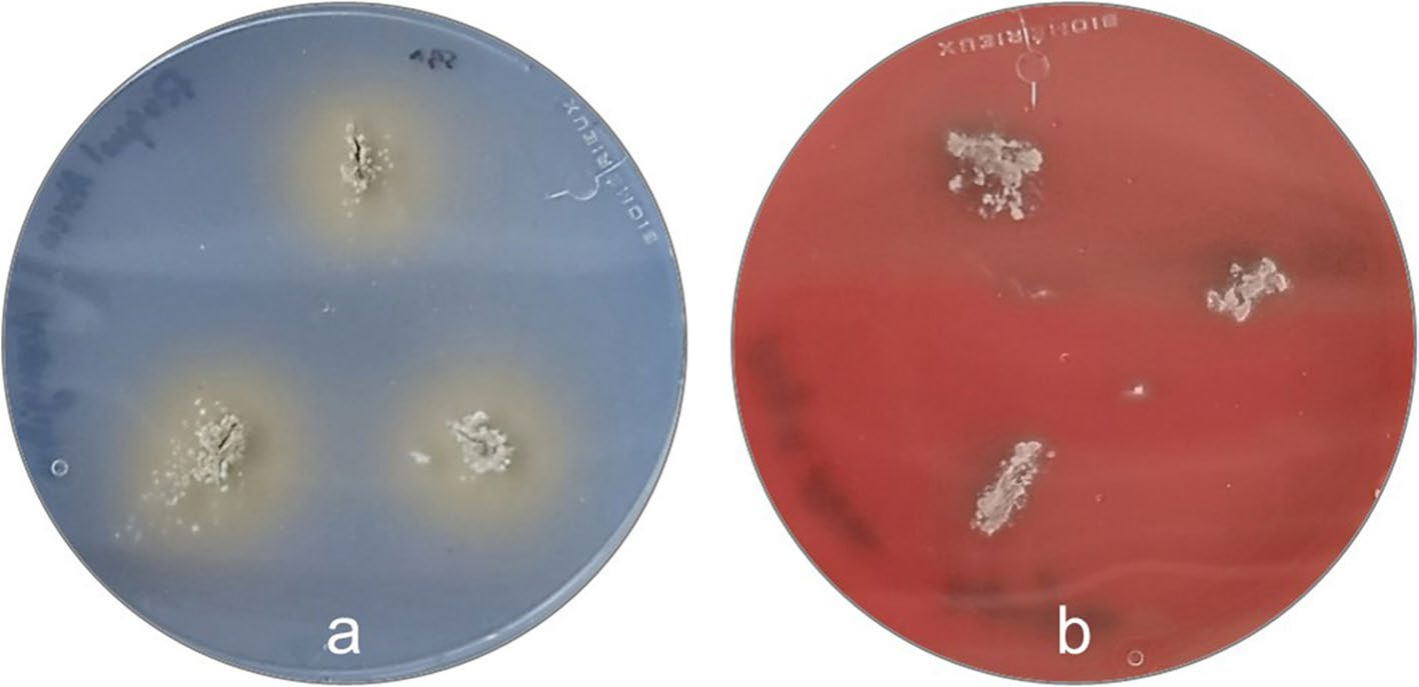
Macroscopic evaluation of isolate’s ability to produce virulence factors. **a** Plate Spirit Blue Agar, where is possible to observe clear areas around the colonies, indicative of lipase production; **b** Plate of Columbia Agar + 5% Sheep Blood, where is possible to observe the activity of hemolysins (α-hemolysis) through the presence of brownish areas around the colonies

The isolate virulence profile was assessed phenotypically by evaluating its ability to form biofilms and to produce several enzymes, including hemolysins, lipase, lecithinase, DNAse, protease and gelatinase, using specific media as shown on Table 1. This isolate was able to produce hemolysins (brownish areas around the colonies), lipase (clear area around the colonies) [30] (Fig. 7) and DNase (pink halo around the colonies) [31], not being able to produce biofilm, lecithinase, protease or gelatinase.

**Table 1:** Phenotypic virulence traits tested and respective media

Phenotypic Virulence Trait	Culture Media	Incubation conditions	Positive result	Reference
Biofilm	Red Congo Agar [BHI Infusion Broth (VWR, Leuven, Belgium), Bacteriologic Agar (VWR, Leuven, Belgium), sucrose and Red Congo Stain (Sigma-Aldrich, St. Louis, USA), in a concentration of 0.0008%]	72 h at 25 °C	Black halo around the colonies	[32]
Hemolysins	Columbia Agar + 5% Sheep Blood (bioMérieux, Marcy-l’Etoile, France)	72 h at 25 °C	Clear halo (β- hemolysis) or green or brown discoloration (α- hemolysis) around the colonies	[30, 33]
Lipase	Spirit Blue Agar (Difco, Detroit, USA), supplemented with 0.25% Tween® 80 and 25% commercial olive oil	24 h at 25 °C	Blue color halo around the colonies	[30, 34]
Lecithinase	Egg Yolk Agar [Tryptic Soy Agar (VWR, Leuven, Belgium), supplemented with 10% egg yolk emulsion (VWR, Leuven, Belgium]	72 h at 25 °C	Clear halo around the colonies	[35]
DNAse	DNAse agar (VWR, Leuven, Belgium) supplemented with toluidine blue	72 h at 25 °C	Pink halo around the colonies	[31]
Protease	Skim Milk Agar [Skim Milk powder (Oxoid, Hampishire, UK) and Bacteriological Agar (VWR, Leuven, Belgium)]	72 h at 25 °C	Clear halo around the colonies	[36]
Gelatinase	Nutrient Gelatin Agar	72 h at 25 °C	Gelatin liquefaction	[30, 37]

The Virulence Index (V. Index) value was determined according to the formula developed by Singh & Ekka [38]:

$$\mathrm V.\mathrm{Index}=\frac{number\;of\;positive\;virulence\;factors}{number\;of\;virulence\;factors\;tested}$$

The isolate presented a virulence index of 0.43 ($3/7$).

The patient was initially submitted to an oral itraconazole treatment, 5 mg/kg q24h for 8 weeks. There were significant improvements in clinical signs at 3 weeks of treatment, after which the animal was able to support the affected limb. A control radiography was taken after 6 weeks of treatment, showing disappearance of the periosteal reaction.

## Discussion and conclusions

The genus *Aspergillus* comprises more than 185 species, being divided in 8 subgenera – Aspergillus, Fumigati, Circundati, Terrei, Nidulates, Ornati, Warcupi and Candidi – and in 22 sections [9]. Among them, *A. fumigatus*, *A. flavus* and *A. niger* are the most frequently isolated species [39]. Others, such as *A. terreus* and *A. versicolor* are less frequent [9, 13, 16, 17].

*A. terreus*, classified in the subgenera Terrei, is the most common species of the subgenera and is found worldwide in various environmental habitats [40]. In veterinary medicine, there are already various reports of disseminated infections caused by *A. terreus* [13–17, 24]. To the author’s knowledge, this is the first report of osteomyelitis caused by *Aspergillus terreus* in a dog in Portugal. Infections by this species have been reported in Spain, Australia, Israel, South Africa and United States of America [12, 24].

In dogs, despite being more frequent in German Shepperd, *A. terreus* infections have already been reported in other breeds, such as Labrador Retriever, Rhodesian Ridgebacks, English Setter, Pug, Labrador Retriever cross, Hound cross, Whippet [8], Dalmatian [13] and Red Cloud Kelpie [14]. This species is an important pathogen because of its intrinsic resistance against amphotericin B [41, 42] and its reduced azole-susceptibility due to target gene over-expression or the presence of efflux pumps [26, 43].

The portal of entry of *Aspergillus* sp. is thought to be via the respiratory tract, through inhalation of spores, with subsequent hematogenous spread if not eliminated by the host immune system [1]. Sites of embolic dissemination of fungi include the kidney, spleen, lymph nodes, bone, heart, lung, eyes, pancreas, bone marrow, brain, urinary bladder, prostate, pleura, adrenal, stomach, uterus, thyroid and thymus, in descending order of prevalence [13]. *A. terreus* has a unique characteristic, which is the capacity of production of spores in the affected tissues, and the hematogenous spread of these spores is probably the cause of infection dissemination [21]. So, while there is no knowledge of the primary infection route in the case reported here, there is a possibility that the pathogen entered the body via inhalation, the gastrointestinal tract or a wound.

Moreover, fungi are able to cause disease and overtake the immune system of the host by producing several virulence factors, which are associated with their pathogenic potential [44]. Virulence factors identified in *Aspergillus* species include the production of biofilms, and hydrolytic enzymes such as hemolysins, proteases, proteinases, lipases, phospholipases, amylases and ribonucleases [19, 20]. Biofilms protect *Aspergillus* from phagocytosis and antifungals action, allowing its exponential growth [45]. Hydrolytic enzymes cause degradation of cells and various tissue molecules, such as proteins, carbohydrates, lipids and phospholipids. As such, they can impair cell function, leading to cell lysis and necrosis, and contributing to the patient clinical status. In fact, production of virulence factors may contribute to the development of invasive infections and their detection can help to adapt therapeutic protocols [46]. The *A. terreus* isolate obtained in this study presented a V. Index of 0.43, which may explain its association with host dissemination and osteomyelitis development.

The few previous reports of disseminated aspergillosis caused by *A. terreus* treated with itraconazole had good improvement of clinical sings. However, in only one report the animal has fully recovered and eliminated the agent [14], and the majority resulted in euthanasia [17]. This is not surprising since the bone tissue characteristics may impair drug diffusion, being sometimes necessary to increase antifungal doses to avoid complications [22] and infection recurrence, which is very frequent [47].

This case report demonstrates that, even if fungal osteomyelitis associated with *A. terreus* are not frequent, they should be considered as a differential diagnosis, especially since an early diagnosis is associated with an increased therapy success. Therefore, further studies are needed to characterize the pathogenic profile of these isolates and to investigate the response to therapy and recovery in cases of disseminated aspergillosis.

## Supplementary Information

**Additional file 1.** ITS1 sequence. Sanger sequencing results using ITS1 primer.

**Additional file 2.** ITS2 sequence. Sanger sequencing results using ITS2 primer.

## Data Availability

The datasets used and/or analyzed during the current study are available from the corresponding author on reasonable request.

## Abbreviations

CT: Computerized Tomography
BHIb: Brain Heart Infusion Broth
H&E: Hematoxylin and Eosin
LCB: Lactophenol Cotton Blue
