# Supplementary material for: Osteomyelitis caused by Aspergillus terreus complex in a dog: a case report
Source: BMC Vet Res. 2023 Jun 8;19:76. doi: 10.1186/s12917-023-03628-x (PMC10249244; doi:10.1186/s12917-023-03628-x)
Supplement: Supplementary file 2 — Additional file 2. ITS2 sequence. Sanger sequencing results using ITS2 primer. [file 12917_2023_3628_MOESM2_ESM.docx]

>PCR00264957+ITS2+GCTGCGTTCTTCATCGATGC 256

nnnnncnngnttganttttactgattgcaaagaatcacactcagactgcaagctttcagaacagggttcatgttggggtctccggcgggcacgggcacgggggcgagtcaccaccagggggtgggaggttgggccataaagacccaaactcagtaaagatccttccccggggtgaggttggcaagtaataacccacactcggtaatgatccttcctcaagttcacctagagaagtccgcaggttcacctacggaag
